# A Personalized Physical Activity Coaching App for Breast Cancer Survivors: Design Process and Early Prototype Testing

**DOI:** 10.2196/17552

**Published:** 2020-07-15

**Authors:** Francisco Monteiro-Guerra, Gabriel Ruiz Signorelli, Shreya Tadas, Enrique Dorronzoro Zubiete, Octavio Rivera Romero, Luis Fernandez-Luque, Brian Caulfield

**Affiliations:** 1 Insight Centre for Data Analytics School of Public Health, Physiotherapy and Sports Science University College Dublin Dublin Ireland; 2 Salumedia Tecnologias Seville Spain; 3 Insight Centre for Data Analytics School of Computer Science University College Dublin Dublin Ireland; 4 Computer Engineering School Universidad de Sevilla Seville Spain

**Keywords:** user-centered design, physical activity, coaching, behavior change, mobile app, mobile phone, breast cancer, usability

## Abstract

**Background:**

Existing evidence supports the many benefits of physical activity (PA) in breast cancer survival. However, few breast cancer survivors adhere to the recommended levels of activity. A PA coaching app that provides personalized feedback, guidance, and motivation to the user might have the potential to engage these individuals in a more active lifestyle, in line with the general recommendations. To develop a successful tool, it is important to involve the end users in the design process and to make theoretically grounded design decisions.

**Objective:**

This study aimed to execute the design process and early prototype evaluation of a personalized PA coaching app for posttreatment breast cancer survivors. In particular, the study explored a design combining behavioral theory and tailored coaching strategies.

**Methods:**

The design process was led by a multidisciplinary team, including technical and health professionals, and involved input from a total of 22 survivors. The process comprised 3 stages. In stage 1, the literature was reviewed and 14 patients were interviewed to understand the needs and considerations of the target population toward PA apps. In stage 2, the global use case for the tool was defined, the features were ideated and refined based on theory, and a digital interactive prototype was created. In stage 3, the prototype went through usability testing with 8 patients and was subjected to quality and behavior change potential evaluations by 2 human-computer interaction experts.

**Results:**

The design process has led to the conceptualization of a personalized coaching app for walking activities that addresses the needs of breast cancer survivors. The main features of the tool include a training plan and schedule, adaptive goal setting, real-time feedback and motivation during walking sessions, activity status through the day, activity history, weekly summary reports, and activity challenges. The system was designed to measure users’ cadence during walking, use this measure to infer their training zone, and provide real-time coaching to control the intensity of the walking sessions. The outcomes from user testing and expert evaluation of the digital prototype were very positive, with scores from the system usability scale, mobile app rating scale, and app behavior change scale of 95 out of 100, 4.6 out of 5, and 15 out of 21, respectively.

**Conclusions:**

Implementing a user-centered design approach for the development and early evaluation of an app brings essential considerations to tailor the solution to the user’s needs and context. In addition, informing the design on behavioral and tailored coaching theories supports the conceptualization of the PA coaching system. This is critical for optimizing the usability, acceptability, and long-term effectiveness of the tool. After successful early in-laboratory testing, the app will be developed and evaluated in a pilot study in a real-world setting.

## Introduction

### Background

According to the current findings, physical activity (PA) is the most well-established lifestyle factor associated with breast cancer survival [1]. Rapidly accumulating research demonstrates that routine exercise throughout and after treatments offers multiple benefits for breast cancer survivors, including prevention of cancer recurrence, mitigation of treatment side effects (such as lymphedema and fatigue), and improvement of their physical function and quality of life [2-4]. Despite the growing evidence supporting PA in breast cancer survivorship, only a minority of these individuals adhere to the recommended levels of PA [5,6]. Novel and engaging strategies are needed to ensure that participants are adhering to PA that is of high enough intensity and frequency to meet the PA recommendations.

Mobile health (mHealth) has emerged as an important tool for health behavior change interventions [7]. Currently, mobile devices can accurately measure PA at any time and place, creating opportunities to provide real-time tailored support and motivation toward an active lifestyle [8]. In line with this, mobile apps for PA coaching have emerged and have been investigated as a platform to motivate people to be active through recommended goals, feedback on activities performed, and potentially enjoyable experiences [9,10]. An increasing body of evidence indicates that these technology-based interventions may be well received by breast cancer survivors and hold promise for PA promotion initiatives [11-13].

An underlying challenge of these technology-supported interventions is the high attrition rates, with users stopping the use of these systems after a few days or weeks [14-16]. Among the factors associated with user abandonment are that apps are largely targeted at generally healthy individuals and do not address the specific needs of the end users [17]. Studies with breast cancer survivors suggest that the direction of PA systems should meet the detailed requirements of this particular population [13,18], who may be less motivated to engage in PA and who face unique barriers to reaching the recommended level of PA [19,20]. These include physical (eg, fatigue, weight gain, and neuropathy), environmental (eg, lack of knowledge, job and family obligations, and weather), and psychosocial (eg, low confidence and emotional imbalance) limitations [19,20]. Related literature supports the use of a user-centered design (UCD) approach, which focuses on the users and their needs and is considered a prerequisite for useful technology being associated with an increase in the success of these systems [21,22]. Furthermore, it is suggested that breast cancer survivors want a PA app experience targeted not only to their needs on a group level but also tailored to each individual user [13,23,24]. In line with this, personalized or tailored coaching mechanisms can be leveraged to help create experiences that are individualized for each user [25,26]. These are believed to influence the user’s attention and contribute to long-term engagement and adherence to these apps [27]. In addition, theory-based behavior change methods have been shown to influence the effectiveness of technology-supported interventions [7] and should be considered in the design of tools that aim to increase PA [8,26].

Currently, a few PA apps reported in the literature target breast cancer survivors [11,24,28,29]; however, these seemed to be in the early stages of development and evaluation and lack proper reporting of design decisions. Only the papers describing the Bounce app presented clear details on the design process, behavioral theory foundations, and features [24,30]. Further research is needed to better understand the design of mobile PA interventions for breast cancer survivors and how to create a more tailored and engaging experience to increase long-term adherence of these users with the coaching systems.

### Objectives

This paper aimed to report on the UCD of a personalized PA coaching app for breast cancer survivors that targets the needs of breast cancer survivors at both the group and individual levels. The system is grounded on existing theory, models, and empirical evidence on personalized coaching, behavior change, and linear progression exercise training. The paper describes the 3 design stages of the tool: (1) user and context research, (2) app conceptualization and early prototyping, and (3) prototype testing.

## Methods

### User-Centered Design Process

A UCD approach was followed. UCD is considered a prerequisite for useful technology and a successful intervention [21,31]. The process involves a multistage, problem-solving process that investigates the needs, desires, and limitations of users to increase the success rate of usability in computerized systems [22].

**Figure 1:**
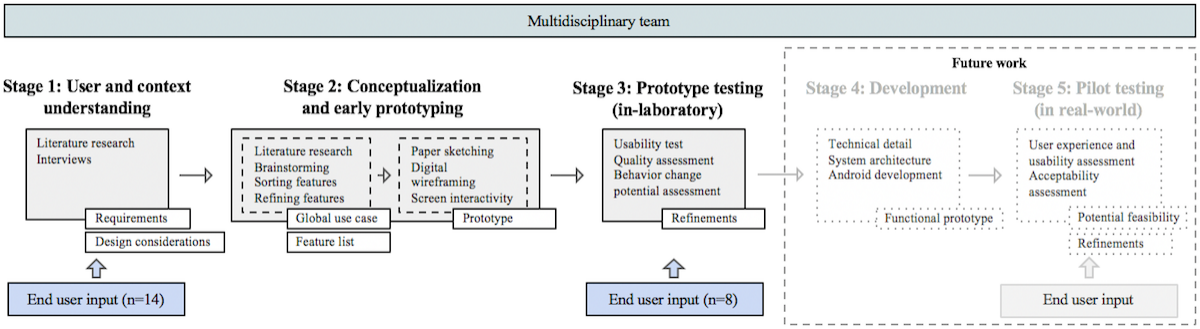
Schematic of the app’s user-centered design process.

The proposed design stages were based on Shah’s methodological framework [31] and on implementations of such an approach in different studies [32,33]. The design process so far involved 3 stages (Figure 1): stage 1—user and context understanding, stage 2—conceptualization and early prototyping, and stage 3—prototype testing. In stages 4 and 5 (future work), a functional prototype will be developed and then pilot tested for potential feasibility in real-life settings.

### Multidisciplinary Team

A multidisciplinary team guided the design process and included 5 members from different backgrounds and expertise: FG, OR, EZ, and ST have an engineering background (biomedical, computer, electronic, and computer science engineering, respectively) and have experience in the fields of digital health apps, human-computer interaction, participatory health informatics, and user experience; GS has a sports science background, with expertise in the field of PA in oncology. Matilde Mora Fernández also has experience in the field of PA in oncology and provided useful insights to the team at different stages of the design process.

### Theoretical Foundation

To guide the design process, we used applicable behavior change theory, with the aim of increasing the long-term effectiveness of the PA system [34]. Appropriate theoretical frameworks (self-determination theory [SDT] [35] and social cognitive theory [SCT] [36,37]) and constructs were identified from related work and empirical evidence to highlight factors, barriers, and determinants that brought important insights into the design of the solution. In addition, to facilitate the design and reporting of theory-based components, the Coventry, Aberdeen, and London-Refined (CALO-RE) taxonomy of behavior change techniques proposed by Michie et al was considered [38]. The CALO-RE taxonomy has been used particularly in PA and healthy eating interventions. It has been reported as a useful tool for researchers to design effective interventions as it provides a more straightforward and simple approach to identifying the factors most likely to create positive effects on PA behavior change [38].

Personalization theory was also explored in the tool design, which may help increase the intended effects of the app communication and, in that way, increase the effectiveness of the behavior change intervention [27]. Op den Akker et al [25] proposed a model for real-time tailoring of PA coaching apps, which defined 7 different methods of personalization. A more recent review by Monteiro-Guerra et al [26] provided a detailed analysis of the existing personalized PA coaching apps and strategies, which provided useful insights for the design of the solution proposed in this work.

### Participant Recruitment for User Studies

The inclusion criteria for research participants were (1) to be oncology patients with a history of breast cancer that finished primary curative treatment (surgery, radiotherapy, and chemotherapy), (2) to be aged more than 18 years, (3) to own and use a mobile phone or smartphone, (4) to have the ability to read and speak Spanish, (5) to have no known impairments or comorbidities, and (6) to have no restrictions on PA. The participants were recruited from a specialized oncology clinic by placing a phone call to the eligible individuals identified in the patient database. Recruitment was conducted until saturation of results was reached, which was considered when there was no new information (themes) arising from the qualitative data.

This study was approved by the Research and Ethics Committee of Junta de Andalucia in Spain. Subjects’ agreement for participation was obtained through an informed consent process.

### Stage 1: User and Context Research

In the first stage, thorough research was performed on the needs and requirements of breast cancer survivors regarding content, expected benefits, features, and personalization and motivational aspects of the proposed tool. For this purpose, we performed (1) a qualitative study with the target users and (2) a review of related literature, from which we identified the consequences for the design of our solution.

Semistructured interviews were conducted with 14 breast cancer survivors. Three of the team members, FG, OR, and EZ, were involved in this study. The interviews involved a combination of open-ended questions on PA adherence and technology interest and a slideshow presentation with examples of PA app features to obtain participants’ thoughts and opinions on the featured content. The interviews were audio-recorded, transcribed verbatim, and analyzed using thematic content analysis [39]. For the purposes of this paper, only an overview of the main insights gathered from the aforementioned qualitative study is presented here.

A rapid literature review was performed on February 2019 to complement the findings from the user study on (1) barriers and facilitators for PA; (2) attitudes, needs, and preferences from breast cancer survivors on PA apps; and (3) information on behavioral theory used in PA interventions for breast cancer. A combination of medical and technological keywords was used in the search string: *breast cancer* AND app* AND *mobile* OR *smartphone* AND physical activit* OR exercis* OR walk*. The searches were performed on 3 web-based databases, PubMED Central, Association for Computing Machinery digital library (ACM), and Scopus, with the use of the selection criteria. Then, a title and abstract review was performed. Other relevant papers were identified through a snowballing process by screening the reference list of the included ones for related papers [40].

### Stage 2: Conceptualization and Early Prototyping

The goal of this phase was to conceptualize the idea of the app, to define a list with potential functionalities, and to create a low-fidelity prototype.

#### Conceptualization

After the analysis of the literature and clinical guidelines, a high-level concept of the tool was proposed, which is defined as the global use case. The global use case was designed to facilitate communication of the idea of the tool to the design team, without giving too much direction to their thoughts [32]. It was defined by the authors FG, OR, EZ, and GS in a design session, based on the knowledge gathered from the literature, and validated by Matilde Mora Fernández, the collaborator expert in PA and breast cancer survivorship. The validation process relied on a discussion with Matilde Mora Fernández, which revised the global use case and provided suggestions to refine it. The global use case described on a high level the main objectives of the tool, the technological platform, and essential requirements.

The ideation process to elicit features for the mobile app consisted of 2 group sessions with FG, OR, EZ, and GS. The first session was introductory, where the results from stage 1 were discussed and clarified to each of the team members. The second session involved feature elicitation, followed by sorting and selecting features. Each session lasted for 45 min to 1 hour. A brainstorming technique was implemented using the affinity wall method [41]. First, the team members were asked to read through the global use case and the list of user needs and requirements, which was made visible to everyone in 1 slide, and then, for 15 min each member individually wrote their ideas for potential features. Once the ideas were submitted, similar ideas were combined, resulting in a preliminary list of features. The criteria-based evaluation [42], which uses a decision matrix, was used to choose the main ideas, based on the considerations from stage 1. The criteria used for feature ranking were based on the strengths, weaknesses, opportunities, and threats analysis [43]. On the basis of the ranking given and team agreement, the top ideas for features were selected.

These ideated features went through a refinement phase, which involved an iterative process of analyzing design considerations from stage 1, to further specify the app functionalities. In this process, 2 researchers (FG and OR) identified a list of requirements and preferences from breast cancer survivors that were related to each of the elicited features. In addition, considerations were taken from the CALO-RE taxonomy [38], which presents 40 techniques and the psychological constructs each purport to change. We have considered the model of personalization in real-time PA coaching apps [25], which proposes 7 concepts or strategies to adjust the different properties of communication to the users. These design considerations were listed and mapped to each of the main features to help refine the app functionalities and how they needed to be designed. Furthermore, we followed the model on linear progression training and the insights from the experts in exercise and cancer to construct the foundations of the training plan for the app.

#### Prototyping

On the basis of the ideas generated, the team started the prototyping phase. This phase consisted of the creation of a digital and interactive low-fidelity prototype, in which priority was given neither to content nor visuals [44]. The wireframes were created by FG using the NinjaMock software and were then transferred to the Proto.io software to simulate the interactions between the buttons and screens. The prototype was then reviewed by the rest of the team, which provided suggestions for improvements through the software. The objective of this low-fidelity prototype was to facilitate idea communication and to perform early evaluations, which were conducted in stage 3 of the design process.

### Stage 3: Prototype Testing

The third stage of the process sought to explore an early evaluation of the system, involving both user and expert testing. The evaluation was directed toward assessing the usability, quality, and behavior change potential of the concept ideated.

#### User Evaluation

Usability relates to the extent to which a system can be used by the end users to achieve specified goals with effectiveness, efficiency, and satisfaction. Indicators for these are error rate, task completion time, and a satisfaction rating questionnaire, respectively [45,46]. In this study, a mixed methods approach was followed for usability testing [47,48]. The study was led by FG and OR. The test was conducted in a laboratory setting, which involved the end users interacting with the prototype created in stage 2. A think-aloud procedure was performed. The participants first completed an initial questionnaire covering demographics, technology use and interest, and PA level. Then, after a short explanation, the participants performed 7 predetermined tasks with the interactive prototype. Participants were asked to verbalize their thoughts while completing the tasks. Interactions with the prototype and the participants’ comments were recorded using the AZ Screen Recorder app. In addition, the researcher FG observed the participants throughout their task performance, registering the user’s comments and suggestions, the number of errors, the task duration, and any indication of needing assistance or confusion. The number of errors was calculated as the number of times there was an erroneous interaction (eg, the task required the user to find the information tab and the user clicked on the profile button). Task duration was calculated from the moment the researcher presented the task until the task was completed, only considering when the task was performed without errors. In addition, participants valued the complexity of each task using the single ease question (SEQ), which is a scale to rate tasks from 1 (very difficult) to 7 (very easy) [49]. All participants completed the Spanish version of the system usability scale (SUS) [50], a 10-item questionnaire used to quickly and accurately assess the usability of a system. Higher scores indicate better usability.

At the end of the session, a short interview was performed to address the users’ understanding of particular features and information provided by the app, the general opinions on the app and its usefulness, and if there was anything missing in the app.

#### Expert Evaluation

An expert evaluation was performed with the mobile app rating scale (MARS) [51] and the app behavior change scale (ABACUS) [52] to assess the quality of the app and the potential for behavior change, respectively. The MARS was used to examine app elements, such as engagement, functionality, utility, aesthetics, and information. This scale includes 23 items across 5 categories, with each item scored using a series of questions on a 5-point ordinal scale response. An overall functionality score out of 5 was derived using this scale. The ABACUS scale comprises 21 items and was used to examine the potential behavior change of the app in relation to goal setting, action planning, barrier identification, self-monitoring, and feedback. Two technical experts, with previous experience using these tools, performed the evaluation of the concept based on the low-fidelity prototypes and a document detailing the app functionalities.

#### Analysis

The SUS, MARS, and ABACUS scores were calculated following the standard procedures for each scale. The SUS mean and SD were calculated for the scores across all participants. In addition, the mean and SD of SEQ ratings and task duration, across all participants, were calculated for each particular task and for all tasks. For each evaluator, a MARS score out of 5 was calculated under 3 of the 4 sections of the scale. Some of the aspects covered by the scale, including the section of design aesthetics, were not considered due to the use of a low-fidelity prototype. The mean of these scores produced an overall score for each evaluator, and the mean of the overall scores for each evaluator provided an overall score for the app quality. The 2 evaluators also identified the presence of ABACUS items under each of the 4 sections of the scale. Discrepancies were discussed between the expert evaluators until an agreement was reached. The number of items identified, out of 21 possible items, was added to provide an overall score for the app’s behavior change potential.

The audio data, from the think-aloud procedure and the short user interviews, were transcribed, anonymized, and translated. The transcripts were, then, analyzed by FG and OR to identify the salient aspects about usability issues, functionalities liked by the users, and suggestions for improvements.

## Results

### Participant Characteristics for User Studies

The first user study (interviews in stage 1) was held with 14 participants and the second (usability in stage 3) with 8 participants. In study 1, the participants’ ages ranged from 43 to 69 years, with a mean of 52.8 (SD 8.8) years, and in study 2, it ranged from 38 to 63 years, with a mean of 48.4 (SD 8.0) years. The number of years since diagnosis ranged from 2 to 11.5, with a mean of 5.2 (SD 2.9) for participants in study 1, and from 0.5 to 4.5, with a mean of 2.3 (SD 1.6) in study 2. In general, participants were educated and employed, were very interested in technology, had ready access to technological devices, and had shown high usage of a variety of technology functionalities. Participants’ access to technology and usage can be found in Multimedia Appendix 1. According to the international physical activity questionnaire-short form [53], most participants had at least a moderate level of PA. However, when looking at the activity type and intensity, 57% (8/14) of participants in study 1 and 50% (4/8) in study 2 did not adhere to the PA guidelines [54]. Participant characteristics are presented in detail in Table 1.

**Table 1:** Participant characteristics.

Characteristic		User study 1 (n=14), n (%)		User study 2 (n=8), n (%)	
**Marital status**					
	Single		4 (29)		N/A^a^
	Married		10 (71)		N/A
	Divorced		0 (0)		N/A
**Education**					
	Basic school		1 (7)		1 (13)
	High school		2 (14)		2 (25)
	Higher education		2 (14)		0 (0)
	University or college		9 (64)		5 (63)
**Current employment status**					
	Not working		3 (21)		1 (13)
	Employed		11 (79)		7 (88)
Receiving pharmacological treatment		10 (71)		8 (100)	
Indication for PA^b^		11 (79)		8 (100)	
**IPAQ-SF^c^ level**					
	High		1 (7)		3 (38)
	Moderate		11 (79)		5 (63)
	Low		2 (14)		0 (0)
Adheres to PA guidelines (>150 min per week=moderate activity or >75 min per week=vigorous activity)^d^		6 (43)		4 (50)	
**Interest in technology**					
	Agree or strongly agree		12 (86)		8 (100)
	Neutral		2 (14)		0 (0)
	Disagree or strongly disagree		0 (0)		0 (0)
**Self-reported skill with technology**					
	Agree or strongly agree		9 (64)		7 (88)
	Neutral		5 (36)		1 (13)
	Disagree or strongly disagree		0 (0)		0 (0)
**"I like to experiment with new technology"**					
	Agree or strongly agree		7 (50)		6 (75)
	Neutral		5 (36)		2 (25)
	Disagree or strongly disagree		2 (14)		0 (0)

^a^N/A: not applicable.

^b^PA: physical activity.

^c^IPAQ-SF: international physical activity questionnaire-short form.

^d^Information inferred from IPAQ-SF answers.

### Stage 1: User and Context Research

#### User Needs, Requirements, and Preferences for Physical Activity Apps

The findings from the qualitative study with 14 breast cancer survivors provided insight on barriers and motivators for PA and opinions on a variety of app-based intervention characteristics. From the literature review, we have included 5 papers to provide a more complete perspective on the barriers and facilitators of PA [19,20,55-57] and 11 papers with at least some information regarding the attitudes, needs, and preferences of breast cancer survivors on PA apps [12,13,18,23,24,29,58-62]. These included papers on user studies with the Bounce app and the Smart After Care app, both designed for breast cancer survivors, which provided relevant insights to the design. An overview of the barriers and facilitators for PA in breast cancer survivors is provided in Textbox 1.

The list of requirements taken from our interviews and related work is presented in Table 2.

**Overview of barriers and facilitators for physical activity in breast cancer survivors.**

- Barriers
  - Lack of time
    - Consequence of job and family responsibilities
  - Lack of confidence
  - Low motivation
  - Physical limitations
    - Fatigue, lymphedema, joint pain, muscular pain, neuropathy, and weight gain
    - Current physical activity (PA) level compared with pretreatment
  - Lack of information/support for PA
    - Lack of information on the type and amount of PA recommended
    - Fear and uncertainty of starting exercising without guidance
  - Misconceptions about PA
    - Fear of potential side effects
  - Emotional imbalance
    - Not feeling good
    - Stress or anxiety
  - Access to facilities
    - Inconvenient timetable or distant location
  - Seasonal weather
- Facilitators
  - Reserve time during the week for PA
  - Knowing and perceiving the benefits
  - Being nudged to be more active
  - Support to overcome insecurities
  - Emotional support
  - Tailored information
  - Prescription of PA by the health care professionals
  - Training plan tailored to their needs and PA level
  - Clear and realistic objectives
  - Quantify activity performed
  - Having an active family
  - Support from family and close friends

**Table 2:** Overview of breast cancer survivors’ requirements and preferences for physical activity apps.

Type	From user study	Literature review—additional insights
General intervention characteristics	Activity monitoring and feedback Preference for information on steps, calories, distance, pace, and duration of activity PA^a^ prescription and goal setting Scheduling tool and activity reminders Tailored experience Progress monitoring and visualization Straightforward representation of activity performed and incremental improvements Preference for daily and weekly progress feedback Information on how their progress translates into physiological processes (eg, benefits for weight management) Simplicity and ease of use Mixed reactions toward a game-like design (eg, points, rewards, avatars, and competitions) Consider strategies to manage emotional challenges (eg, encourage connecting with counselor and include relaxation and meditation exercises).	Preference for walking activities (most appealing and main form of exercise) Other liked activities included resistance training and yoga Feedback on time spent in various intensities of activity Evidence-based content Attractive design Friendly graphic displays Possibility to integrate with wearable activity trackers Preference for more straightforward representations of numerical data (compared with having gamified themes) Suggestions for integration of a Newsfeed Information on benefits and harms/risks of exercise for breast cancer survivors
Personalized experience	Adaptive activity plan and goals Progressive but attainable goals Customizable exercises and exercise schedule Targeting user characteristics (eg, age, treatment types, and preferences) Sensitive to PA level and physical limitations/injuries Individualized progress feedback Targeting user’s situational/external context (eg, weather and location) Personalized recommendations Interface simulating a virtual coach	Incremental levels adjusted to the user experience Change intensity or amount of exercise program to reflect user’s improvement Ensures correct execution of exercises Target value-based goals Suggestions for integration of a symptom tracker Suggestions for tracking energy level, how they feel, and sleep quality
Positive communication	Motivation/encouragement Encouraging prompts during activity Recognition for achievements Positive tone Absence of pressure Just enough reminders and notifications	Casual and concise tone Motivational messages
Social connectedness (varied opinions)	Involvement of family and close friends Mixed reactions toward connecting with other peers (eg, social networking, competitions, or ability to see others’ progress) Ability to connect with a professional (eg, a psychooncologist and/or an exercise trainer)	Role-model narratives Preference for a more private experience
Trustworthiness	Transparent data privacy and security Developed with and validated by clinical/health experts Include contact information of people involved in the app development	N/A^b^
Data sharing and portability	Optional and customizable data sharing Willingness to share with health care professionals	Ability to keep an electronic record of their workouts on the app Ability to download data to a PC^c^ Extensive yet passive data collection

^a^PA: physical activity.

^b^N/A: not applicable.

^c^PC: personal computer.

#### Behavioral Theory for Physical Activity Interventions in Breast Cancer

From searching the literature for appropriate behavioral foundations for design, there seems to be little consensus on optimal theories and theory integration techniques to change PA behaviors in this population [63-65]. According to some studies, the transtheoretical model of behavior change, SCT, and SDT are the most appropriate models for behavioral interventions for breast cancer survivors [2,18]. In our qualitative study, the findings suggest that the motivational factors and determinants of PA adherence in breast cancer survivors are in line with the constructs of SCT (self-efficacy and expected outcomes) and SDT (competence, autonomy, and social relationships). Together with the CALO-RE taxonomy of behavior change, these insights will facilitate the integration and reporting of behavior change techniques in our solution. The psychological mediators identified in our user study and associated CALO-RE techniques are presented in Multimedia Appendix 2.

### Stage 2: Conceptualization and Early Prototyping

#### Global Use Case

The high-level concept of the tool is defined as a real-time coaching system to support and motivate breast cancer survivors to increase adherence to PA; that is smartphone-based; that simulates the interactions with a PA coach; that ensures that users engage in activities of high enough intensity to meet the PA recommendations; that provides a tailored experience, on a population level and on an individual level; that focuses on walking activities; and that is based on the PA guidelines and recommendations for breast cancer survivors. The ultimate goal of the tool is to help breast cancer survivors reach and maintain the recommended levels of PA.

#### App Functionalities

One of the main steps of this stage 2 was the ideation process, which resulted in the specification of 7 main app features and their respective refined subfeatures (see Table 3). An extended version of the table, including design considerations from stage 1 and from the CALO-RE taxonomy used for feature refinement, is presented in Multimedia Appendix 3.

**Figure 2:**
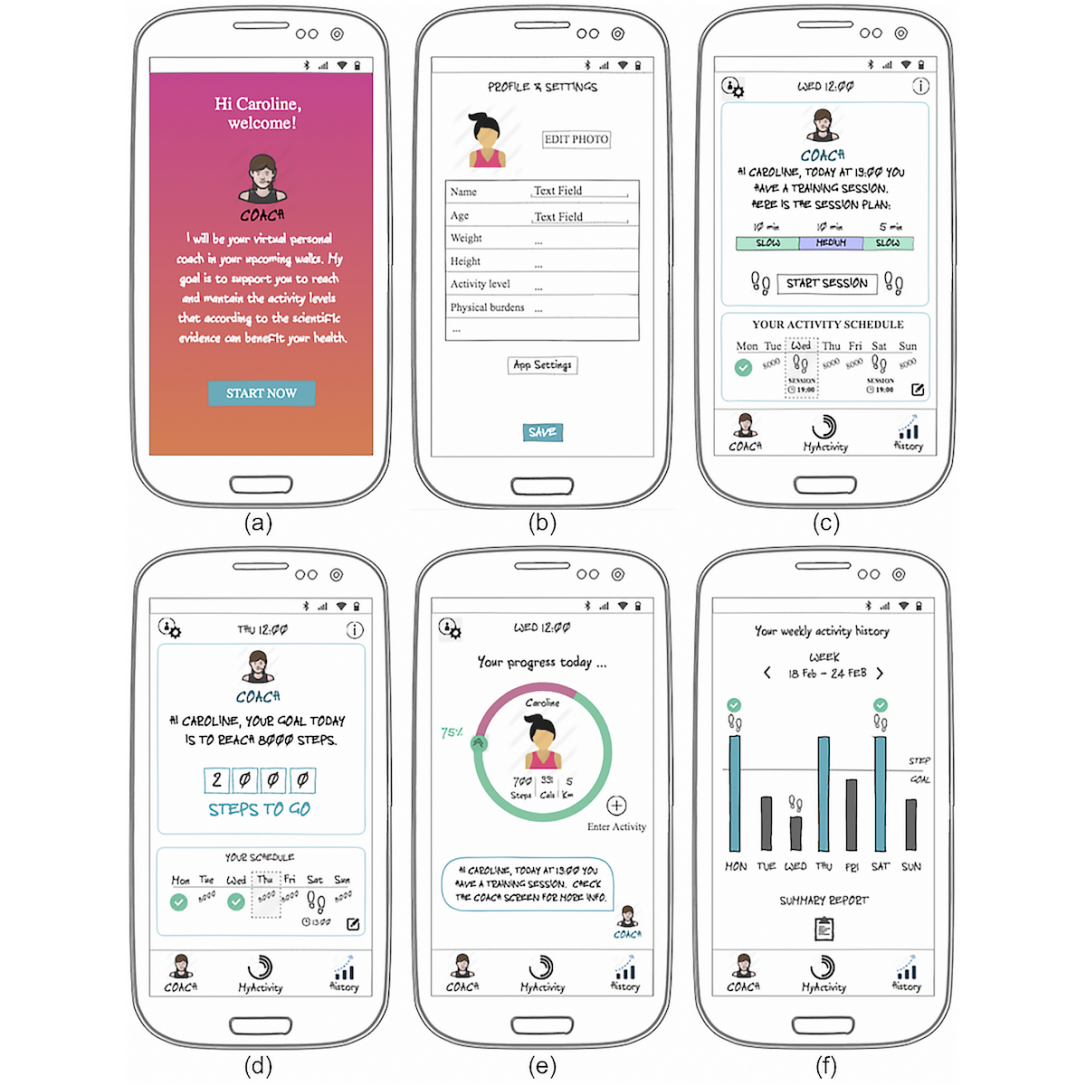
Screenshots of the main prototype screens. (a) Welcome screen; (b) profile screen; (c) coach (main) screen on session day; (d) coach (main) screen on a step goal day; (e) MyActivity screen, with information on the user’s current activity status; and (f) history screen, with information on past activity and access to the weekly summary reports.

**Figure 3:**
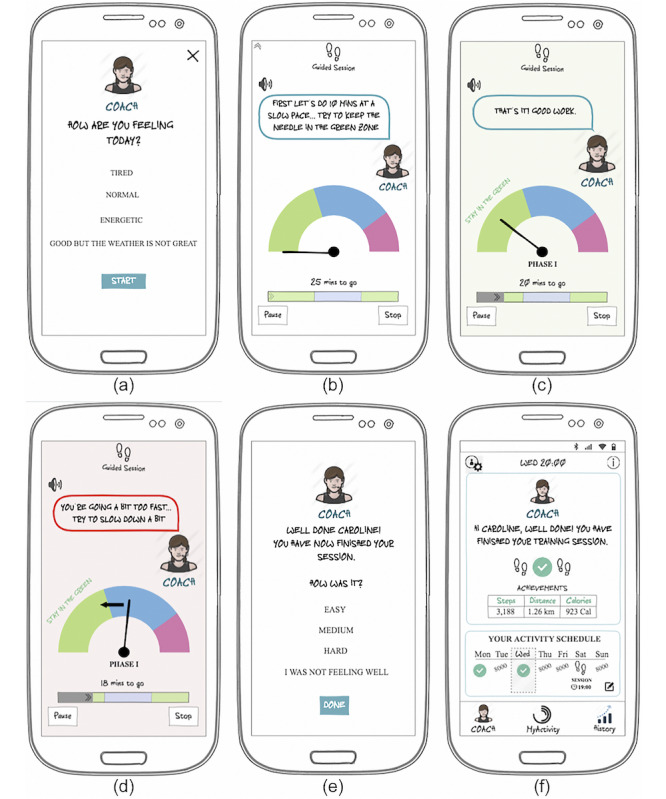
Screenshots of the simulation of a guided session. (a) Presession perceived tiredness, (b) example instruction for session phase duration and pace, (c) example cue to keep up the pace, (d) example cue to slow down the pace to the ideal zone of the current phase, (e) postsession perceived session difficulty, and (f) session achievements in the coach screen.

Details on the PA program and the personalized coaching system are provided in the following sections. The interactive low-fidelity mock-ups (translated from Spanish to English) are shown in Figures 2 and 3, including the main screens and the guided session simulation, respectively.

**Table 3:** List of app functionalities.

Main features (ideated)	General description (ideated)	Subfeatures (refined based on considerations from stage 1 and the CALO-RE^a^ constructs)
Training plan	Activity program guided by the PA^b^ guidelines and recommendations for breast cancer survivors; based on linear progression training; with adaptable levels; visually represented by an activity schedule; includes reminders for activity.	Information about PA program, guidelines and potential benefits for the users; a plan that sets the number of activities per week and its duration and difficulty; a weekly activity schedule; baseline assessment for current PA level; assessment of perceived difficulty; adjustable plan level; push notifications and reminders for activities scheduled; push notifications and reminders to review the plan and reschedule activities.
Adaptive goal setting	Activity objectives adjusted to the user.	Present the user with clear daily objectives in the main screen; set achievable but challenging goals; progress bars; inform of long-term benefits of achieving goals; present automatic adaptation to the user’s profile information, progress, user’s perceived fatigue and perceived difficulty; notifications of goal adjustments; weekly goal adjustment.
Real-time monitoring, feedback, and motivation during activity sessions	In-session or ”workout” coaching; visual and easy to understand; combines real-time monitoring, feedback and motivation.	Predefined walking sessions; guidance to meet the session plan; intuitive interface to provide session information; shows the session progress (time); shows the user’s pace in real time through a glanceable visual display; sends cues to control the pace; provides positive reinforcement and recognition; coaching cues are in textual and audio format; shows achievements when the session is concluded with a breakdown of the session: steps taken; calories burned; distance walked and session duration.
Activity status through the day	Feedback on the total activity performed until that point in the day and progress toward the daily goal.	Screen with numeric representations of active time: steps taken, calories burned and distance walked; progress bar showing progress toward the daily goal; option to manually entry activity; encouraging pop-up messages.
Activity history	Tracking past activity; graphic display; simple and intuitive.	History screen; bar chart representation of daily activity in relation to the goal; week-by-week information.
Periodic summary reports	Descriptive summary of the activity performed during the week and the overall progress in the program; tips for improvement; motivation to be active and to follow the program.	Weekly activity reports; presents a breakdown of the activity performed during the week; bar chart representation comparing current week activity with previous weeks; communicates progress in the plan; encourages users to follow the program; provides tips according to the user’s physical barriers; informs users of PA benefits.
Challenges^c^	Unexpected activity challenges.	—^d^

^a^CALO-RE: Coventry, Aberdeen, and London—Refined taxonomy.

^b^PA: physical activity.

^c^To be considered in future iterations of the prototype.

^d^Not available.

#### Physical Activity Program

From the evidence on the topic of PA and breast cancer, it is known that the beneficial effects associated with PA are more pronounced with moderate or vigorous intensity compared with mild intensity physical activities [66]. Breast cancer survivors are recommended to perform at least 120 to 150 min per week of moderate to vigorous activity [54]. This means that a common activity such as walking, which breast cancer survivors have a general preference for, if done at the right intensity and for the right time, can be sufficient to reach the minimum recommended levels of PA. Furthermore, walking is an activity that people can do everywhere and that nowadays can be easily monitored using smartphone technology. Hence, a training program was developed, which targets walking activities and aims to guide these individuals to reach and maintain the amount and intensity of activity recommended in the guidelines.

The activity program was based on the model of linear progression training to allow for difficulty adjustment according to the user’s actual PA level [67]. It will be integrated into a future functional version of the prototype in the form of a training plan. Adjustments will be made session by session, and involve, first, changing the session volume and, in the following session, changing the intensity level. There will be different difficulty levels in the plan: beginner, intermediate, adapted, and advanced. The activity plan will include 3 guided training sessions per week (with controlled intensity), with a minimum duration of 30 min per session. In addition, the user will be encouraged to reach a daily step goal that is adjusted weekly. The objectives in the plan will be gradually more challenging and adjusted regularly. Figure 2 depicts how these training specifications will be represented in the coach tab. 

On the basis of the work of Tudor-Locke et al [68], walking cadence or walking pace will be measured to infer the training zone (intensity) of the activity being performed. Hence, based on this and on linear progression training, the training format will be defined as a number of training phases or traits with a certain duration, each on a specific training zone inferred from the walking pace—slow, medium, or fast (see example of session format in the coach tab screenshot, Figure 2).

From the training program specifications, some of the tool features were refined (eg, the coach tab and schedule, in Figure 2, and the real-time guided sessions to control the user’s walking pace, in Figure 3).

#### Personalized Coaching System

The results from stage 1 highlighted the importance of exploring a personalized coaching experience. Therefore, the coaching system in the app will combine a number of strategies proposed in the model of real-time personalization in PA coaching apps. The different characteristics of the app that will be considered for the creation of a personalized experience are described below.

#### Virtual Coach

A human-like interface between the user and the app will simulate the interactions with a real coach (see virtual coach representations, Figures 2 and 3). It will serve as a sender or source of all the communication provided by the system through a variety of forms: general information, support, tips, activity feedback, motivation, and summary reports. It will provide clear, concise, and positive communication. Such a strategy is expected to increase the feeling of trust and credibility, increase motivation, and increase the feeling of personalized experience and interaction

#### Targeted Feedback

The app will provide feedback on the activities of the user (number of steps, distance, and calories burned), estimate calories spent by the user based on the user’s characteristics, inform the user of their daily personalized goals and progress toward their goal, integrate the user’s name in the communication provided by the coach, provide feedback in a weekly summary report considering the user’s progress in current and past weeks, and provide tips according to the user’s physical burdens.

#### Adaptive Activity Plan and Goals

Because these individuals vary in PA level and progress and, particularly in this population, there might be physical constraints due to side effects of both cancer and treatment, the app will integrate a rule-based module that adjusts the step goal and training session objectives in line with the model of linear progression training. This will be done considering the user’s baseline level, progress, the user’s perceived fatigue level (presession), and the user’s perceived session difficulty level (postsession).

#### Real-Time Training Session Guidance

This feature will allow to coach users through the guided sessions, in real time. The coach instructional messages or cues will be designed to be clear, concise, and positive, using short and easy messages, and related to the visual content. It will provide feedback on the user’s pace in relation to the ideal pace set for each of the session phases, sending cues to slow down, speed up or to keep pace, inform on time progress through the different phases and the whole session, and give positive reinforcement. Regarding representation, the feedback will be presented in textual, audio, and visual forms. The visual form will use a glanceable display (representing a speedometer) that will provide feedback on the current user’s pace. The needle will represent the current user cadence and each color zone at a certain pace (slow—green, moderate—blue, and fast—pink). In addition, a progress bar will be used to indicate the current time, total session time, and progress through the different training phases (including the demarcated color zones). Figure 3 depicts an example real-time guided session simulation.

### Stage 3: Prototype Testing

This stage of the process sought to explore an early evaluation of the interactive mock-ups, involving both user and expert testing. The evaluation was directed toward assessing the usability, quality, and behavior change potential of the concept ideated.

#### User Evaluation

In the task-based session, participants’ overall results on the posttask SEQ questionnaire revealed that all tasks were easy or extremely easy to complete, with a mean of 6.6 out of 7 (SD 0.5). Participants’ mean duration in completing the tasks was 4.6 (SD 1.2) seconds. The overall mean number of task errors was 0.3 (SD 0.4). Participants suggested that if they had previous practice, they would have easily completed the tasks without errors. Table 4 gives further details on the task-based session results.

The mean SUS score was 95 (SD 6.3) out of 100, which can be considered above average (above a score of 68). The score obtained can be converted to a percentile rank above 95%, which is interpreted as grade A+. This means that the prototype has higher perceived usability than 95% of all products tested with this scale (Multimedia Appendix 4).

From the short usability interviews, overall, the participants were very satisfied with the usability of the app, finding it very intuitive and easy to use. Furthermore, some participants suggested that an app like this would help them to get motivated to do PA, find time to do more activity, serve as a companion, provide support, and make them comply with the PA plan. In addition, participants reported some usability problems and suggested a number of improvements or additional app features. The information synthesized from the short interviews is presented in Textbox 2.

**Table 4:** Task-based session results.

Tasks	Task description	Participants with errors, n (%)	Errors, mean (SD)	Completion time in seconds, mean (SD)	SEQ^a^ score, mean (SD)	Observations
Task 1	Which steps should you take to get to the profile screen?	4 (50)	1.0 (1.1)	4.8 (1.8)	6.5 (0.5)	This was the task with more user errors. Participants suggested the task was easy and that errors were associated with a lack of attention (eg, not taking a look at the whole screen before interacting with the prototype) or due to the lack of a first interaction experience with the prototype.
Task 2	Which steps should you take to find the amount of activity you have done so far today?	2 (25)	0.4 (0.7)	4.1 (3.1)	6.3 (1.2)	A generally easy to complete task. Errors were associated with participants going to the History tab instead of the MyActivity tab. This confusion originated from the ambiguity on how the task was posed, which made both answers correct in some way. In addition, it seemed that the purpose of the MyActivity tab was only clear after having a first look at it.
Task 3	Which steps should you take to reschedule an activity session from Friday to Saturday?	2 (25)	0.5 (0.9)	6.5 (4.3)	5.8 (1.0)	This task had the lowest SEQ^a^ score and was the one that took longer to complete. Participants suggested that finding the rescheduling button was easy, but the process of setting the new schedule was not clear.
Task 4	Which steps should you take to start and finish a guided session?	1 (13)	0.1 (0.4)	4.6 (2.7)	6.6 (0.5)	A generally easy task. Error due to lack of attention (eg, not taking a look at the whole screen before interacting with the prototype) or due to the lack of a first interaction experience with the prototype. Participant pressed the MyActivity tab instead of the *Start Session* button. In addition, in the session simulation, some participants thought the green color was for fast pace and pink for slow pace, when in reality it was the opposite.
Task 5	Which steps should you take to find the activity you have done so far this week?	0 (0)	0.0 (0.0)	2.5 (0.8)	7.0 (0.0)	Very easy. No issues.
Task 6	Which steps should you take to find your activity history from last week?	0 (0)	0.0 (0.0)	5.0 (2.3)	7.0 (0.0)	Very easy. No issues.
Task 7	Which steps should you take to find the user manual?	1 (13)	0.1 (0.4)	5.0 (3.3)	6.8 (0.4)	Considered very easy. Error was associated with a lack of attention (eg, not taking a look at the whole screen before interacting with the prototype) or due to the lack of a first interaction experience with the prototype.

^a^SEQ: single ease question.

**Aspects highlighted by participants in the task-based session and interviews.**

- Usability issues
  - The session plan phases understood as different session alternatives.
  - Issue with understanding the purpose of MyActivity tab.
  - The scheduling process not clear.
  - The purpose of the session icon in the history tab, to distinguish session days from step goal days, is not clear.
  - Green and pink zones of the speedometers associated with the opposite pace zones (eg, some participants thought green was for fast pace and pink for slow pace).
  - Issue associating the current screen to the corresponding buttons at the bottom.
- Positive perceptions
  - Easy to use and straightforward.
  - Guided session.
  - Prescription of an activity plan.
  - Quantification of activity, knowing calories spent, distance, and steps.
  - Validation by professionals, based on evidence.
  - Access to activity history.
  - May motivate the user to comply with the training.
  - May serve as a companion.
  - May reinforce motivation for physical activity.
- Suggestions for improvements
  - Change text font.
  - Improve graphic design.
  - Include feedback information on time spent walking.
  - Include recommendations and examples of resistance exercises.
  - Include optional short training.
  - Include ‘free training’, the option to do guided sessions on days without a scheduled session.
  - Include the option to download data to the computer to view annual progress.
  - Induce the user to relate the app and physical activity as another part of the treatment, but one that is fun.
  - Include audio feedback during the guided sessions.
  - Include training or an explanation of how the app works (user manual or guide).
  - Include reminders for activity.
  - Include sleep and weight tracking.

#### Expert Evaluation

Although not all aspects from the MARS could be addressed, because of the low-fidelity design of the prototype at this stage, the app quality mean score was 4.6 (SD 0.4) out of 5. In particular, for each of the sections on engagement, functionality, and information, the app mean scores were 4.2 (SD 0.6), 4.8 (SD 0.4), and 4.9 (SD 0.1) out of 5, respectively (Multimedia Appendix 5). Regarding the ABACUS scale, the app scored 15 out of 21 possible behavior change techniques (Multimedia Appendix 6). These results suggest good quality and potential for behavior change of the proposed concept.

## Discussion

### Principal Findings

In this study, we describe a UCD process for the development of a PA coaching app for breast cancer survivors. To our knowledge, this is the first work combining, and reporting in detail, the use of behavioral theory, personalized coaching strategies, and linear progression training for the design of a PA app for breast cancer. The design team gathered the user requirements and insights from experts in exercise and cancer and translated these into the concept of the solution. The concept was refined based on the theoretical foundations, and its viability was confirmed by the technical members of the team. An interactive low-fidelity prototype of the tool was created and assessed in both user and expert evaluations. The user-centered process provided insight into the needs and preferences of the end users on PA apps, which will increase the likelihood of success of the proposed solution.

This tool was designed to simulate the interactions with an exercise coach. It includes an adaptive walking regimen and a number of personalized coaching features that aim to support and motivate users to adhere to it, with the ultimate goal of helping them to progressively reach and maintain the recommended levels of activity for breast cancer survivors. The main functionalities of the system include a training plan and schedule, adaptive goal setting, real-time feedback and motivation during walking sessions, activity status through the day, activity history, weekly summary reports, and activity challenges. One of the main features of the concept proposed is the ability to provide live coaching during the guided walking sessions, monitoring the user’s cadence (through the built-in sensors in the phone), and providing real-time guidance and encouragement to keep the pace within the ideal training zones and to follow the session plan. This particular strategy ensures that users are doing an activity of high enough intensity and duration to meet the PA recommendations.

With regard to personalization, the app aims to (1) provide an automatic and reliable way of providing adaptive training and (2) provide personalized coaching for each user, considering a variety of individualization factors that include the users’ personal characteristics (eg, name, age, height, and weight), physical burdens or barriers, baseline PA level, progress, perceived fatigue level, and perceived difficulty. For this purpose, a number of personalization strategies were used, which included a combination of feedback, user targeting, goal setting, and self-learning [26]. Other factors for individualization, including the user’s preferences, routine, and the external context (location and weather), will be considered for integration in upcoming iterations of the solution.

The concept covers, at least in some form, 21 out of the 40 behavior change techniques defined in the CALO-RE taxonomy [38]. Some techniques were identified for possible inclusion in future iterations of the concept (eg, C25—involve a written agreement, C27 and C33—prompt self-talk, and C36—encourage stress management), and others were excluded as they were not in line with the user requirements and context (eg, C28—facilitate social comparison and C32—fear arousal). The use of this taxonomy helped in the concept design and in specifying the behavior change components of the tool, which may facilitate future reporting and evaluation of a technology-based PA intervention.

The results from prototype testing with users and experts were promising, with high scores for usability, quality, and behavior change potential. Several considerations can be taken from these evaluations to inform the future refinement of the prototype, which include further exploring engagement strategies, particularly related to entertainment, customization, and interactivity, and to consider other behavior change techniques, such as providing the ability to export data from the app, to suggest restructuring social or physical environment, and to assist with distraction or avoidance. Other system functionalities that might be considered in the future are, for example, to include resistance exercises in the activity program and app, to share experiences with close friends or family members, and to enable data sharing with health care professionals.

### Comparison With Previous Work

There is a growing trend in the design of PA coaching systems that are aimed at individuals with chronic conditions. The solution presented here attempts to address the particular needs and requirements of breast cancer survivors for PA apps by taking a UCD approach for the conceptualization of the tool. Few are the systems in the literature designed with such a purpose, with only 4 apps identified that had some component of PA coaching for these individuals [23,24,28,29]. Compared with such apps, our solution targets aerobic exercise, specifically walking, which has been suggested as the preferred activity for these individuals. This allowed us to explore a design that addressed in detail the particular user requirements and preferences associated with such activity type. Although the integration of a component for resistance training might be considered in the future, we believe that it would necessarily bring other considerations for design and different implementation requirements, some of which have already been reported in previous work [24]. Another differentiating factor of this work relies on the attempt to create a personalized app experience for these users, a need that was reported recurrently in related literature on PA apps in breast cancer survivorship. Furthermore, we used the CALO-RE taxonomy, which provided important considerations for the integration of behavior change techniques in the tool design.

Despite some similarities in app functionalities compared with those reported for other populations, the solution proposed in this work differs in some ways and has characteristics that are more particular for breast cancer survivors. For example, studies on a healthy population [69,70] and for people with osteoarthritis [71] have highlighted the importance of social and game-like features (eg, competition). In a study with multiple sclerosis, participants were also interested in gamification and wanted, particularly, a tool focused on fatigue management [72]. Our requirements for breast cancer survivors were not directed toward playful and social experiences or fatigue management, which is similar to the findings of a study on chronic obstructive pulmonary disease patients [73]. Besides, our concept has some characteristics that are more specific to the needs and preferences of our target population, which include a tool for managing time for PA (scheduling feature); a PA program, monitoring and feedback targeted specifically at walking activities; and a personalization system that considers the user’s individual characteristics and progress to adjust training and communication provided by a virtual coach.

Related literature reports on key design requirements for a successful PA coaching solution. For example, Consolvo et al [74] demonstrated the importance of giving users proper credit for activities, providing personal awareness of the activity level, supporting social influence, and considering the practical constraints of users’ lifestyles. Bielik et al [75] built on those recommendations and highlighted that these systems should also ensure fair play, provide a variety of motivational tools, provide feedback on activities done, provide short-term and long-term motivation, provide the possibility of integration with existing solutions, and protect users’ privacy. Our concept of the tool seems to be in line with most of these recommendations. The design requirement related to the user’s privacy is an aspect that also came up in the user research stage and that will need to be carefully considered in the development stage, particularly considering the level of personalization proposed for this system. In addition, as stated by Matthews et al [76], better system credibility support features (eg, trustworthiness, expertise, authority, third-party endorsement, and verifiability) need to be incorporated in PA mobile apps if they aim to achieve the highest level of persuasiveness. Such characteristics were also highlighted in our findings from the initial stage of design. Hence, we believe that pointing out to the users that this app was created in collaboration with other breast cancer survivors and professionals, having content based on the evidence, and simulating the interactions with an activity coach, might increase their trust and, therefore, their interest in the system.

Little is known about how to translate and apply the many high-level theory and design recommendations to these systems. In this paper, we have tried to present in a clear and practical way the UCD of a mobile PA coaching app based on foundations from behavior change and personalization constructs. We expect this to facilitate future research on the design of mHealth solutions, particularly PA coaching systems.

### Limitations

The results of the studies with the end users should be considered with caution because of the small sample size. To complement the results of the qualitative study, which included 14 participants, we looked into the literature and compared it with related work. Regarding usability assessment, it is known that tests with 5 participants are able to uncover 85% of usability issues [77,78]. In our study, we had the involvement of 8 participants, and therefore, we think most usability issues have been revealed.

The system evaluation was performed with a low-fidelity prototype, which might have limited the outcomes from the testing stage in some way. For example, interaction of the user with the prototype was limited to the main screen interactions, which did not allow a real simulation of the functionalities provided by the app. In addition, the analysis of some of the testing scales was restricted to the current stage of the prototype. On the other hand, the fact that we have performed such comprehensive early testing provided us with important insights that will feed the next stages of the design and development process.

### Future Work

From the concept created in this work, we have started the development process of a functional prototype. The next steps involve more iterations on the concept and further feature refinement to detail the app content, integrate the PA program, integrate an appealing graphic design, and perform further system evaluation. Once the functional prototype is developed, a 2- to 3-week pilot study with breast cancer survivors will be performed to assess potential acceptability, usability, and feasibility.

### Conclusions

Centering the design on the end users, breast cancer survivors, and their context revealed valuable requirements and considerations to be taken into account for the design of a tool that aims to address their specific PA needs and motivate these individuals to increase their PA levels. This is essential for increasing the usability and acceptability of the tool. Furthermore, informing the process on the theory and constructs used in tailored PA coaching interventions provided important design insights, which may contribute to the effectiveness, long-term adherence, and acceptability of the system.

At this stage, we have confirmed good usability, quality, and behavior change potential of the prototype in a laboratory setting. A functional prototype will further be tested in pilot and feasibility studies in a real-world environment before it can go through more controlled trials evaluating long-term effectiveness.

This paper details the UCD process for a PA coaching app for breast cancer survivors, which may inform other researchers and developers working in similar mHealth tools.

## Appendix

Multimedia Appendix 1 Participants’ access to technology and technology usage.

Multimedia Appendix 2 Table with considerations on psychological mediators of physical activity adherence in breast cancer survivors associated with Coventry, Aberdeen, and London—refined taxonomy constructs.

Multimedia Appendix 3 Extended version of Table 3 with list of app functionalities and related design consequences from stage 1 and from the Coventry, Aberdeen, and London—Refined taxonomy.

Multimedia Appendix 4 Table with system usability scale results.

Multimedia Appendix 5 Table with mobile app rating scale results.

Multimedia Appendix 6 Table with app behavior change scale results.

## Abbreviations

ABACUS: app behavior change scale
CALO-RE: Coventry, Aberdeen, and London—Refined taxonomy
MARS: mobile app rating scale
mHealth: mobile health
PA: physical activity
SCT: social cognitive theory
SDT: self-determination theory
SEQ: single ease question
SUS: system usability scale
UCD: user-centered design
