# Supplementary figures and images for: A Personalized Physical Activity Coaching App for Breast Cancer Survivors: Design Process and Early Prototype Testing
Source: JMIR Mhealth Uhealth. 2020 Jul 15;8(7):e17552. doi: 10.2196/17552 (PMC7391671; doi:10.2196/17552)

Participants’ access to technology and technology usage


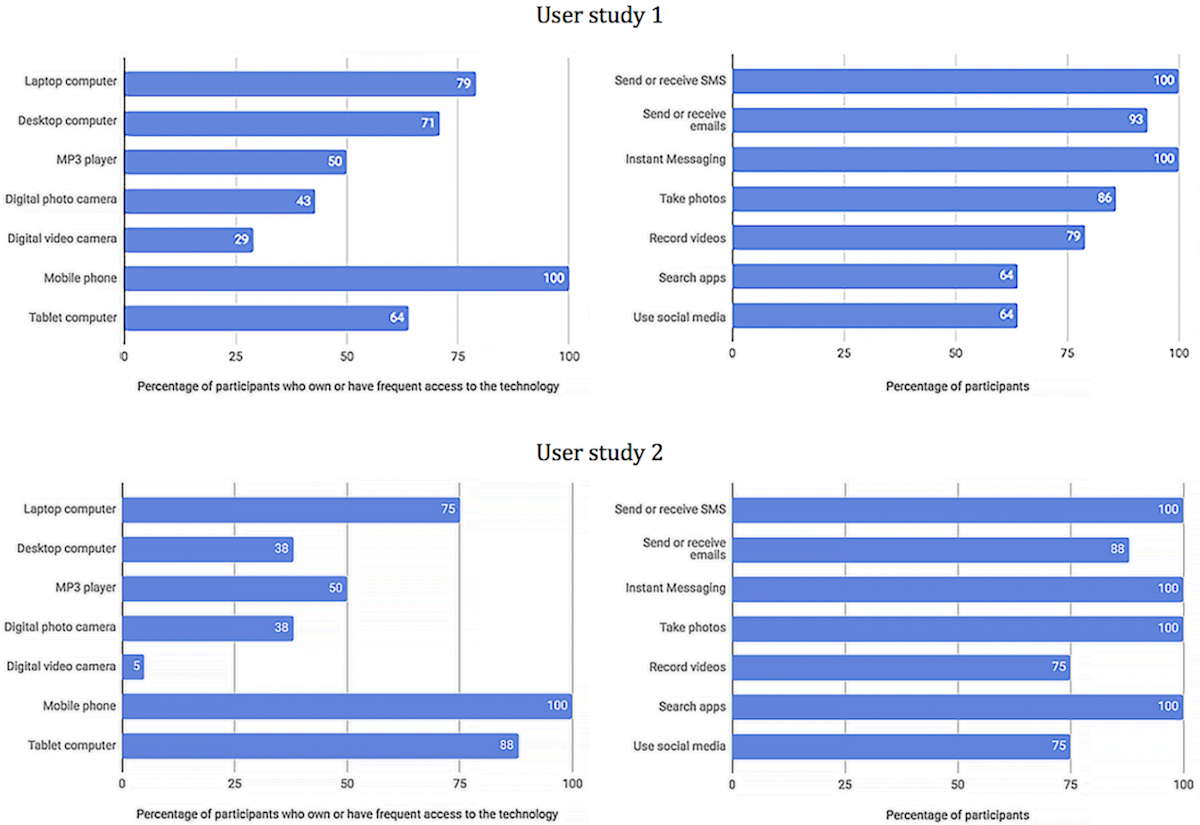

Supplement: Multimedia Appendix 1 [file mhealth_v8i7e17552_app1.docx]
