# Supplementary material for: A Personalized Physical Activity Coaching App for Breast Cancer Survivors: Design Process and Early Prototype Testing
Source: JMIR Mhealth Uhealth. 2020 Jul 15;8(7):e17552. doi: 10.2196/17552 (PMC7391671; doi:10.2196/17552)
Supplement: Multimedia Appendix 2 [file mhealth_v8i7e17552_app2.docx]

Considerations on psychological mediators of physical activity adherence in BCa survivors and associated CALO-RE constructs

| **Psychological Mediators** | **General Description** | **Summary of related insights from interviews** | **Associated CALO-RE Constructs** |
| --- | --- | --- | --- |
| Autonomy | Sense of control or freedom over the behavior and goals. Need to self-regulate one’s experiences and actions. Acting in accordance to one’s goals and values. | Participants highlighted the importance of having a sense of ownership and values alignment with their self-management and PA^a^ experiences. They wanted to have an active role in such experiences because they valued the positive influence it could have in their own and their families’ lives. Also, they wanted to feel in control of their PA experience by self-monitoring, setting a strategy and goals. Absence of pressure towards achieving such goals was also preferred by participants. | C1 and C2 - Provide information on consequences of adhering to the PA plan in general, for BCa survivors, and specific to the individual; C6 - Encourage users to set a general goal achieved through increasing PA (eg, reduce/maintain weight, reduce chances of cancer recurrence, increase QoL); C7 - Involves detailed planning of what the user will do, which includes the frequency and duration of the action plan (eg, activity plan).  Other constructs identified in relation with competence, relatedness and self-regulation can also influence perceived autonomy. |
| Self-efficacy/ confidence | Confidence in ability to take action and persist in action. | Participants’ belief in their capacity to do more PA seemed to be often limited by disease- and treatment-related physical and psychological barriers. Particularly, they have referred to their difficulties in re-starting PA after treatment. In this line, participants highlighted the importance of perceiving improvement and success in their experiences. Also, participants’ source of motivation often came from constructive self-thinking. Suggested app characteristics included positive reinforcement, acknowledgement, individualized progress information, adjusting the PA experience to their personal characteristics (eg, PA level, limitations), and trusting the technology. Another barrier identified that could affect their confidence towards being more active was the lack of PA information. Participants suggested the inclusion of a “prescribed” activity program that would provide detail on the type, amount and duration of PA.  These strategies are also associated with characteristics of competence-support. | C7 - Involves detailed planning of what the user will do, which includes the frequency and duration of the action plan (eg, activity plan); C8 - Task individuals to identify possible barriers to performance and solutions to overcome them. This presumes having an initial plan to increase PA; C12 - Prompt praise or rewards for attempts at achieving an activity goal (not contingent on actual success); C13 - Providing praise, encouragement, rewards for successful performance in completion of activity goal; C18 - Prompting the person to think about past success; C19 - Provide feedback on performance of physical activity, which may include commenting, supporting or critiquing; C21 - Instruction on how to effectively perform the behavior; C22 - Showing the person how to perform the activity through physical or visual means; C33 - Prompt self-talk, as verbal encouragement and support to maintain action; C36 - Encourage to focus on reducing stress and improving emotional control (eg, support from counselor or provide relaxation exercises). |
| Competence and growth | Knowledge and skills necessary to carry out behavior. A gradual improvement in competence is thought to increase self-efficacy. | Participants reported having lower capabilities to perform PA than before treatment, but they also suggested that doing PA made them feel physical and psychological improvements, which increased their sense of competence. They wanted to be provided with appropriate challenges, to have dynamic difficulty of goals (eg, adjusted to their current PA level and progress), and to receive positive feedback and rewards based on effort. Also, app simplicity and ease of use were highlighted by participants and support a sense of competence in using the technology.  Game-like strategies for providing ongoing competence satisfaction were perceived by most as childish and had mixed reactions from participants. However, having different levels of “experience” was positively received and if points were used in that sense, it could improve their perception of it. Rewards were only perceived as useful if they could be exchanged for real experiences (eg, a yoga session). | C9 - Set graded tasks (eg, inclusion of small goals to accomplish an increase in PA level, inclusion of progressive levels and sublevels in the activity plan); C12 - Prompt praise or rewards for attempts at achieving an activity goal (not contingent on actual success); C13 - Providing praise, encouragement, rewards for successful performance in completion of activity goal; C14 - Graded and progressive use of contingent rewards over time; C15 - Encourage to try the target behavior in another situation; C19 - Provide feedback on performance of physical activity, which may include commenting, supporting or critiquing; C21 - Instruction on how to effectively perform the behavior; C22 - Showing the person how to perform the activity through physical or visual means; C27 - Intervention components are gradually reduced in intensity, duration and frequency over time (eg, notifications, reminders), as the person becomes better at performing the behavior. |
| Relatedness/ social support | Sense of being connected to others. | There were varied reactions towards interacting with other survivors, with participants often suggesting wanting social aspects to be optional if included in an app. On the positive side some participants commented that it could help to share and compare experiences and results, and be recognized by others, and to avoid getting “bored” while doing PA. Social features mentioned included having a type of social network and to have a common shared goal among users. On the negative side participants mentioned it could have a negative impact on their emotions (eg, causing feelings of depression) as people often tend to speak about their cancer experiences, which are often negative and sad. Also, group heterogeneity in terms of treatment stage or PA level was reported as barrier to relatedness. To some participants it simply did not suit them and preferred a more private app experience. Involvement of related ones in the PA experience was positively perceived by participants. It was also suggested that it could be dependent on the moment/stage of the cancer journey. | C3 - Provide information about others’ approval (eg, remind users that family and friends will be very supportive of them adhering to a PA program); C4 - Provide information about what others typically do with respect to physical activity (eg, other BCa survivors); C24 – Prompt users to make changes to in their environment in order to facilitate changes in behavior (eg, informing family members so that they may help prompt PA).  Other social techniques, particularly the ones more related to connecting with other users, were not addressed in the current concept proposed. |
| Outcome expectancies | Beliefs about likely results of action. | Awareness of PA’s physical and psychological benefits was identified by participants as a source of motivation to be more active and could be explored by an intervention to support self-efficacy. | C1 and C2 - Provide information on consequences of adhering to the PA plan in general, for BCa survivors, and specific to the individual; C40 - Encourage the user to consider future rewards associated with increasing PA (eg, getting access to other levels or other plans, access to other exercises, or simply to consider the possible gains of exercise). |
| Self-regulation | Goal-setting, planning, and monitoring tasks to achieve behavior change. | Participants were very motivated by being able to monitor a variety of variables related to their PA (eg, number of steps, calories spent, speed). They suggested that the more information one has on the performed activity, the better. Also, they wanted to have activity objectives defined for the short-term (eg, daily and weekly objectives) and some for the long-term, if not too much on the long-term. These goals should be progressively more challenging. Participants also suggested the inclusion of a training plan and scheduling tool for goal setting-support, which would help them resume and maintain PA. | C5 - Encourage to begin and maintain the change towards increasing the activity level (eg, include sub-goals or preparatory behaviors, include daily goals/activity taks); C6 - Encourage users to set a general goal achieved through increasing PA (eg, reduce/maintain weight, reduce chances of cancer recurrence, increase QoL); C7 - Involves detailed planning of what the user will do, which includes the frequency and duration of the action plan (eg, activity plan); C8 - Task individuals to identify possible barriers to performance and solutions to overcome them. This presumes having an initial plan to increase PA; C9 - Set graded tasks (eg, inclusion of small goals to accomplish an increase in PA level, inclusion of progressive levels and sublevels in the activity plan); C10 - Prompt review of goals and the extent to which they were achieved; C16 – Involves keeping a detailed record of activity; C17 – As C16, but focused on measurable outcomes (eg, blood pressure and/or weigh reduction); C19 - Provide feedback on performance of physical activity, which may include commenting, supporting or critiquing; C20 - Provide information of where and when to perform the activity; C23 - Teach to use cues such as alerts/reminders to prompt users to initiate their PA routine; C25 - Involve a written agreement on the resolution of the user to follow the activity plan, which is witnessed by others (eg, healthcare professionals or relatives); C27 - Use of follow-up prompts (eg, reminders, notifications) to remind user to keep a started PA routine; C35 - Relates to C8, but this one applies when the behavior has already been changed. It is about planning how to maintain the behavior and avoid relapse (eg, encourage to do the activity at home if bad weather is a possible barrier); C38 - Support in time management (eg, inclusion of activity schedule). |

^a^ PA: Physical activity.
