# Supplementary material for: A Personalized Physical Activity Coaching App for Breast Cancer Survivors: Design Process and Early Prototype Testing
Source: JMIR Mhealth Uhealth. 2020 Jul 15;8(7):e17552. doi: 10.2196/17552 (PMC7391671; doi:10.2196/17552)
Supplement: Multimedia Appendix 3 [file mhealth_v8i7e17552_app3.docx]

Extended version of Table 3 with list of app functionalities and related design consequences from stage 1 and from the Coventry, Aberdeen, and London—Refined taxonomy.

| Main features | General description | Design considerations from stage 1 | CALO-RE^a^ constructs | Subfeatures (refined) |
| --- | --- | --- | --- | --- |
| Training plan | Activity program guided by the PA^b^ guidelines and recommendations for breast cancer survivors; based on linear progression training; with adaptable levels; visually represented by an activity schedule; includes reminders for activity. | Should allow the user to visualize the plan for the week ahead; should provide a range of activities with a variety of intensity levels and length; should be sensitive to user-identified information (eg, personal health considerations, age, physical limitations, current PA level, user’s progress); should have a certain degree of customization in rescheduling; could learn from user’s routine to discern times of day users usually exercise^d^; should send the just enough periodic notifications and reminders, at opportune times, to comply with plan. | C1 and C2—Provide information on consequences of adhering to the PA plan in general, for breast cancer survivors, and specific to the individual; C5—Encourage user to make a behavioral resolution or a preparatory behavior (eg, achieve or maintain the recommended levels of PA or buy equipment); C6—Encourage users to set a general goal achieved through increasing PA (eg, reduce or maintain weight, reduce chances of cancer recurrence, increase QoL^c^); C7—Involves detailed planning of what the user will do, which includes the frequency and duration of the action plan (eg, activity plan); C9—Set graded tasks (eg, inclusion of small goals to accomplish an increase in PA level, inclusion of progressive levels and sublevels in the activity plan); C20—Provide information of where and when to perform the activity; C23—Teach to use cues such as alerts or reminders to prompt users to initiate their PA routine; C38—Support in time management (eg, inclusion of activity schedule); C40—Encourage the user to consider future rewards associated with increasing PA (eg, getting access to other levels or other plans, access to other exercises, or simply to consider the feeling of satisfaction for being more active). | Information about PA program, guidelines and potential benefits for the users; a plan that sets the number of activities per week and its duration and difficulty; a weekly activity schedule; baseline assessment for current PA level; assessment of perceived difficulty; adjustable plan level; push notifications and reminders for activities scheduled; push notifications and reminders to review the plan and reschedule activities. |
| Adaptive goal setting | Activity objectives adjusted to the user. | Must offer short-term goals to achieve long-term goals that bring value to the user; must show progress indicators toward short-term goals; goals should be attainable; should be automatically recommended by the app; short term should be considered as a week; increments in difficulty should be based on the weekly activity progress; app should allow users to input personally held goals^d^; goals should be adapted to the users’ characteristics (age, treatment, physical limitations, personal health concerns, PA preferences^d^, PA levels), and trends over time; should detect user’s physical problems and adapt its functions; app must not decrease the goals due to previous unachieved goals. | C5—Encourage to begin and maintain the change toward increasing the activity level (eg, include subgoals or preparatory behaviors, include daily goals or activity tasks); C6—Encourage users to set a general goal achieved through increasing PA (eg, reduce or maintain weight, reduce chances of cancer recurrence, increase QoL); C9—Set graded tasks (eg, gradually more difficult activity goals). | Present the user with clear daily objectives in the main screen; set achievable but challenging goals; progress bars; inform of long-term benefits of achieving goals; present automatic adaptation to the user’s profile information, progress, user’s perceived fatigue and perceived difficulty; notifications of goal adjustments; weekly goal adjustment. |
| Real-time monitoring, feedback, and motivation during activity sessions | In-session or ”workout” coaching; visual and easy to understand; combines real-time monitoring, feedback and motivation. | Should provide a straightforward way to monitor progress; should include visual representations; should recognize and congratulate when users achieve something; should provide positive reinforcement; should have a casual, concise, and positive tone; could have an encouraging voice during exercise. | C12—Prompt praise or rewards for attempts at achieving an activity goal (not contingent on actual success); C13—Providing praise, encouragement, rewards for successful performance in completion of activity goal; C15—Encourage to try the target behavior in another situation; C19—Provide feedback on performance of PA, which may include commenting, supporting or critiquing; C21—Instruction on how to effectively perform the activity; C22—Showing the person how to perform the activity through physical or visual means. | Predefined walking sessions; guidance to meet the session plan; intuitive interface to provide session information; shows the session progress (time); shows the user’s pace in real time through a glanceable visual display; sends cues to control the pace; provides positive reinforcement and recognition; coaching cues are in textual and audio format; shows achievements when the session is concluded with a breakdown of the session: steps taken; calories burned; distance walked and session duration. |
| Activity status through the day | Feedback on the total activity performed until that point in the day and progress toward the daily goal. | Should give straightforward representation with numeric values; should include visual representations of distance, calories, steps and active time; should show progress toward the goal; should provide positive reinforcement; should feedback users on how exercise performed is influencing them; could include feedback on sedentary time^d^; should give the option to manually add activity. | C13—Providing praise, encouragement, rewards for successful performance in completion of activity goal; C19—Provide feedback on performance of PA, which may include commenting, supporting or critiquing. | Screen with numeric representations of active time: steps taken, calories burned and distance walked; progress bar showing progress toward the daily goal; option to manually entry activity; encouraging pop-up messages. |
| Activity history | Tracking past activity; graphic display; simple and intuitive. | Must provide users with rich data visualization allowing them to compare their weekly progression; should show weekly data. | C10—Prompt review of goals and the extent to which they were achieved; C16—Involves keeping a detailed record of activity; C18—Prompting the person to think about past success; C19—Provide feedback on data about their own recorded activity. | History screen; bar chart representation of daily activity in relation to the goal; week-by-week information. |
| Periodic summary reports | Descriptive summary of the activity performed during the week and the overall progress in the program; tips for improvement; motivation to be active and to follow the program. | Should send weekly summaries on progress through the week; should show the performance change from week to week; should provide simple but rich data visuals; should translate information into physiological processes. | C1 and C2—Provide information on consequences of adhering to the training plan and PA in general, for breast cancer survivors, and specific to the individual; C3—Provide information about others’ approval (eg, remind users that family and friends will be very supportive of them adhering to a PA program); C5—Encouragement to maintain behavior change, without involving precise planning (eg, a goal may be to exercise more next week); C10—Prompt review of goals and the extent to which they were achieved; C12—Prompt praise or rewards for attempts at achieving an activity goal (not contingent on actual success); C13—Providing praise, encouragement, rewards for successful performance in completion of activity goal; C18—Prompting the person to think about past success; C21—Instruction on how to effectively perform the activity; C35—Relates to C8, but this one applies when the behavior has already been changed. It is about planning how to maintain the behavior and avoid relapse (eg, encourage to do the activity at home if bad weather is a possible barrier); C40—Create anticipation of future rewards (eg, getting people to consider possible gains of exercise). | Weekly activity reports; presents a breakdown of the activity performed during the week; bar chart representation comparing current week activity with previous weeks; communicates progress in the plan; encourages users to follow the program; provides tips according to the user’s physical barriers; informs users of PA benefits. |
| Challenges^e^ | Unexpected activity challenges. | Should be sensitive to the users’ level; should be optional; should be occasional. | —^e^ | — |

^a^CALO-RE: Coventry, Aberdeen, and London—Refined taxonomy.

^b^PA: physical activity.

^c^QoL^:^ quality of life.

^d^To be considered in future iterations of the prototype.

^e^Information not available.
