# Supplementary material for: A Personalized Physical Activity Coaching App for Breast Cancer Survivors: Design Process and Early Prototype Testing
Source: JMIR Mhealth Uhealth. 2020 Jul 15;8(7):e17552. doi: 10.2196/17552 (PMC7391671; doi:10.2196/17552)
Supplement: Multimedia Appendix 4 [file mhealth_v8i7e17552_app4.docx]

SUS results

| SUS Item | Mean (SD)^a^ |
| --- | --- |
| 1. I think that I would like to use this system frequently. | 4.50 (0.76) |
| 2. I found the system unnecessarily complex. | 1.00 (0.00) |
| 3. I thought the system was easy to use. | 4.75 (0.46) |
| 4. I think that I would need the support of a technical person to be able to use this system. | 1.00 (0.00) |
| 5. I found the various functions in this system were well integrated. | 4.50 (0.76) |
| 6. I thought there was too much inconsistency in this system. | 1.38 (1.06) |
| 7. I would imagine that most people would learn to use this system very quickly. | 5.00 (0.00) |
| 8. I found the system very cumbersome to use. | 1.00 (0.00) |
| 9. I felt very confident using the system. | 4.63 (0.52) |
| 10. I needed to learn a lot of things before I could get going with this system. | 1.00 (0.00) |
| Overall system usability score ^b^ | 95 (6.27) |

^a^ Responses for each item on a scale of 1 (totally disagree) to 5 (totally agree).

^b^ Overall score calculated after normalization and transformation of scores for each item, according to the SUS standard procedure.
