# Supplementary material for: A Personalized Physical Activity Coaching App for Breast Cancer Survivors: Design Process and Early Prototype Testing
Source: JMIR Mhealth Uhealth. 2020 Jul 15;8(7):e17552. doi: 10.2196/17552 (PMC7391671; doi:10.2196/17552)
Supplement: Multimedia Appendix 5 [file mhealth_v8i7e17552_app5.docx]

MARS results

| MARS Item | |  | Mean (SD)^a^ |
| --- | --- | --- | --- |
| **Section A: Engagement** | |  |  |
|  | Entertainment | | 4.5 (0.7) |
|  | Interest | | 4.5 (0.7) |
|  | Customization | | 3.5 (0.7) |
|  | Interactivity | | 3.5 (0.7) |
|  | Target group | | 5.0 (0.0) |
| Engagement mean score | |  | 4.2 (0.6) |
| **Section B: Functionality** | |  |  |
|  | Performance | | N/A |
|  | Ease of Use | | 4.5 (0.7) |
|  | Navigation | | 5.0 (0.0) |
|  | Gestural design | | N/A |
| Functionality mean score | |  | 4.8 (0.4) |
| **Section C: Aesthetics** | |  | N/A |
| **Section D: Information** | |  |  |
|  | Accuracy of app description | | N/A |
|  | Goals | | 5.0 (0.0) |
|  | Quality of information | | 5.0 (0.0) |
|  | Quantity of information | | 4.5 (0.7) |
|  | Visual information | | 5.0 (0.0) |
|  | Credibility | | 5.0 (0.0) |
|  | Evidence based | | N/A |
| Information mean score | |  | 4.9 (0.1) |
| Overall quality mean score | |  | 4.6 (0.4) |

^a^ Out of 5. Responses for each item on a scale of 1 (inadequate) to 5 (excellent). N/A (not applicable) was used if app component was not relevant due to the early stage of the prototype.
