# Supplementary material for: A Personalized Physical Activity Coaching App for Breast Cancer Survivors: Design Process and Early Prototype Testing
Source: JMIR Mhealth Uhealth. 2020 Jul 15;8(7):e17552. doi: 10.2196/17552 (PMC7391671; doi:10.2196/17552)
Supplement: Multimedia Appendix 6 [file mhealth_v8i7e17552_app6.docx]

ABACUS results

| ABACUS Item | |  | Final Rating ^a^ |
| --- | --- | --- | --- |
| **Knowledge and information** | |  |  |
|  | 1.1 Does the app have the ability to customize and personalize some features? | | 1 |
|  | 1.2 Was the app created with expertise and/or Does the app provide information that is consistent with national guidelines? | | 1 |
|  | 1.3 Does the app ask for baseline information? | | 1 |
|  | 1.4 Does the app provide instruction on how to perform the behavior? | | 1 |
|  | 1.5 Does the app provide information about the consequences of continuing and/or discontinuing behavior? | | 1 |
| **Goals and planning** | |  |  |
|  | 2.1 Does the app ask for willingness for behavior change? | | 0 |
|  | 2.2 Does the app allow for the setting of goals? | | 1 |
|  | 2.3 Does the app have the ability to review goals, update, and change when necessary? | | 1 |
| **Feedback and monitoring** | |  |  |
|  | 3.1 Does the app give the user the ability to quickly and easily understand the difference between current action and future goals? | | 1 |
|  | 3.2 Does the app have the ability to allow the user to easily self-monitor behavior? | | 1 |
|  | 3.3 Does the app have the ability to share behaviors with others (including social media or forums) and/or allow for social comparison? | | 0 |
|  | 3.4 Does the app have the ability to give the user feedback—either from a person or automatically? | | 1 |
|  | 3.5 Does the app have the ability to export data from app? | | 0 |
|  | 3.6 Does the app provide a material or social reward or incentive? | | 1 |
|  | 3.7 Does the app provide general encouragement? | | 1 |
| **Actions** | |  |  |
|  | 4.1 Does the app have reminders and/or prompts or cues for activity? | | 1 |
|  | 4.2 Does the app encourage positive habit formation? | | 1 |
|  | 4.3 Does the app allow or encourage for practice or rehearsal, in addition to daily activities? | | 1 |
|  | 4.4 Does the app provide opportunity to plan for barriers? | | 0 |
|  | 4.5 Does the app assist with or suggest restructuring the physical or social environment? | | 0 |
|  | 4.6 Does the app assists with distraction or avoidance? | | 0 |
| Total score | |  | 15 |

^a^ Final rating after consensus. 1 – Yes and 2 – No.
